# Synergistic CO_2_ Mineralization and Performance Optimization of FA-CS-PG Ternary Solid Waste System

**DOI:** 10.3390/ma19102145

**Published:** 2026-05-20

**Authors:** Jiayao Zhang, Qingping Wang, Zhiwei Cheng, Luyao Wang

**Affiliations:** 1 School of Materials Science and Engineering, Anhui University of Science and Technology, Huainan 232001, China; z17352180500@163.com (J.Z.); czwxym@163.com (Z.C.); 18655792876@163.com (L.W.); 2 State Key Laboratory for Safe Mining of Deep Coal Resources and Environment Protection, Huainan 232001, China; 3 Anhui Industrial Generic Technology Research Center for New Materials from Coal-Based Solid Wastes, Huainan 232001, China

**Keywords:** industrial solid waste utilization, synergistic effect, CO_2_ mineralization rate, response surface methodology, performance optimization, pore structure

## Abstract

In recent years, there has been an urgent need for integrated solutions to synergistically manage industrial solid waste stockpiling and CO_2_ emissions. Single-component solid waste mineralization, such as those using only fly ash (FA) or carbide slag (CS), often encounters performance bottlenecks, typically characterized by a compressive strength of less than 2 MPa and a carbonation efficiency of under 10%. Furthermore, a systematic quantitative understanding of the synergistic interactions within multi-component systems remains absent. This study employs Response Surface Methodology to investigate the interactive effects of solid waste ratios, the water-to-solid ratio, and alkali content, aiming to elucidate the synergistic mineralization mechanism and overcome the bottlenecks of single solid waste mineralization. Under optimized conditions—specifically, 34% CS, 30% phosphogypsum (PG), a water-to-solid ratio of 0.48, and an alkali content of 27%—the system achieved a 7-day compressive strength of 3.5 MPa and a CO_2_ mineralization efficiency of approximately 16%, representing a significant improvement over typical single solid waste mineralization materials. Microstructural and spectroscopic analyses indicate that CS serves a dual function as both a calcium source for CaCO_3_ precipitation and an alkaline activator for FA. FA constructs a dense aluminosilicate network via pozzolanic reactions, while SO_4_^2−^ released from PG promotes the formation of ettringite, facilitating efficient pore filling and early strength development. Additionally, it was observed that surface pores were filled with more products compared to the interior, forming a gradient pore structure that is dense on the outside and sparse on the inside. The AFt and silicate gel were identified as the key microstructural driver for the performance enhancement. This study not only explores the ternary synergistic mechanism of FA, CS, and PG but also provides a viable pathway for developing high-performance solid waste-based mineralization materials that combine mechanical properties with efficient CO_2_ sequestration.

## 1. Introduction

The global carbon emission crisis poses a severe challenge to climate stability [1]. In 2023, energy-related carbon dioxide emissions reached 37.4 billion tons, exacerbating the greenhouse effect and triggering ecological imbalances [2]. Carbon Capture, Utilization, and Storage (CCUS) technology has emerged as a key solution to mitigate this problem—by capturing CO_2_ from industrial sources or the atmosphere, and then utilizing it as a resource or permanently storing it [3]. The International Energy Agency (IEA) notes that by 2050, CCUS technology could contribute 15% of the global cumulative emission reductions, making it an indispensable component of the carbon neutrality pathway [4]. The application of this technology has a global reach. For instance, in North America, Carbon Cure technology has been deployed in real-world engineering projects, such as the 300 N. LaSalle Tower in Chicago, with the aim of achieving carbon-neutral concrete [5]. In Southeast Asia, Thailand is currently evaluating the feasibility of sequestering CO_2_ emissions from the Mae Moh coal-fired power plant into nearby unmineable coal seams [6]. Currently, a Canadian research team is investigating the CO_2_ sequestration potential of serpentinized ultramafic rocks. They found that CO_2_ can be sequestered in a dissolved phase; however, its practical application faces several challenges, such as low reactivity, slow mineralization rates, and the high costs associated with large-scale mining and transportation [7]. In contrast, industrial alkaline solid wastes, as by-products of industrial production, are widely available and low-cost, enabling a “waste–treats–waste” resource cycle. Among them, FA, CS, and PG [8,9,10,11] are regarded as the most promising raw materials due to their global annual production exceeding 100 million tons and their richness in active components for CO_2_ storage, sparking growing research interest.

While progress has been made in mineralization technologies for single-source industrial solid wastes, a significant challenge remains the trade-off between high carbon sequestration capacity and high energy consumption [12,13]. Zhe et al. [14] evaluated the mineralization performance of FA and achieved a maximum carbon sequestration capacity of 50.3 g/kg under experimental conditions of a liquid-to-solid ratio of 15:1 and a CO_2_ pressure of 4 bar. The study noted that the inherently low calcium content of FA limits its theoretical carbon sequestration capacity. Although pretreatment methods such as ball milling or triethanolamine-assisted mass transfer [15,16,17] can enhance this capacity to 81.44 g/kg, they entail a 42% increase in associated energy consumption [18]. This trade-off underscores the economic bottleneck of relying solely on pretreatment for performance enhancement. In contrast, CS, rich in highly reactive calcium hydroxide, achieves rapid carbonation at room temperature under a CO_2_ atmosphere [19]. However, CS mineralization is hampered by a noticeable product encapsulation effect [20]. Ma [21] revealed that as the liquid-to-solid ratio increases from 1:100 to 10:100, the calcium carbonate crystals transformed from cubic blocks into flaky aggregates, with the D_90_ particle size decreasing from 47.75 μm to 14.90 μm. This dense coating layer forms a mass transfer barrier around the unreacted core, hindering the further progression of the carbonation reaction [22,23]. This phenomenon indicates that the advantage of CS’s high reactivity is difficult to sustain, as the reaction kinetics become severely limited in the later stages. PG faces more fundamental challenges due to the high lattice stability of calcium sulfate [24,25,26,27]. The conversion reaction of phosphogypsum under alkaline conditions is shown in Equation (1), which has an equilibrium constant of only 1.2 × 10^−3^ [28]. This value provides a thermodynamic explanation for the fundamental cause of its poor reactivity. Furthermore, phosphorus impurities further inhibit its reactivity [29]. Even with pretreatment methods such as water or acid washing [30,31,32], overcoming the kinetic limitations remains difficult.

(1) $${\mathrm{CaSO}}_{4}\cdot 2H_{2}O+2\mathrm{NaOH}\;\to \;{\mathrm{Ca}\left(\mathrm{OH}\right)}_{2}+{\mathrm{Na}}_{2}{\mathrm{SO}}_{4}+2H_{2}O$$

To address these limitations, research focus has shifted toward synergistic mineralization using multi-source solid wastes. However, existing studies have predominantly concentrated on binary systems, which exhibit notable shortcomings and lack systematic exploration of ternary synergies. Zhang et al. [33] investigated an FA-CS composite system and found that adding 30 wt% CS promoted uniform nucleation and growth of calcium carbonate crystals on the FA surface, increasing the system’s carbon sequestration capacity by approximately 50% compared with that of single CS. Although this system leverages the aluminosilicate framework of FA and the high calcium reactivity of CS, the complete absence of a sulfate component restricts the formation of ettringite (AFt), which is detrimental to early strength development. In alkali-activated FA-PG systems, Feng [34] observed that incorporating 10% PG promoted the generation of AFt and C-(A)-S-H. The resulting network structure filled pores, enhanced water resistance, and reduced alkali activator consumption by 30%. However, such PG-containing systems often rely on slag or steel slag to provide alkalinity, failing to fully utilize CS as a stronger and more economical alkali source, and insufficiently exploring the synergistic potential between CS and PG. In summary, a research gap remains regarding the FA-CS-PG ternary synergistic system, which is capable of simultaneously integrating the advantages of high-calcium alkalinity, aluminosilicate frameworks, and sulfate sources. Based on this, this study selects the FA-CS-PG ternary system as the research object. This system is expected to achieve complementary advantages: CS provides a highly reactive calcium source and a strong alkaline environment, driving rapid mineralization and activating FA and PG; the micro-aggregate effect and aluminosilicate components of FA can alleviate the product encapsulation of CS and provide a skeleton for late-stage strength; the sulfate ions introduced by PG can react with the aluminum phases of CS and FA to generate AFt, enhancing early strength and consuming PG.

To validate this hypothesis, this study employs Response Surface Methodology (RSM) to systematically investigate the interactive effects among multiple factors, including solid waste proportion, water-to-solid ratio, and alkali content. Combined with microstructural analysis, the synergistic mechanisms between components are revealed. By establishing predictive models for CO_2_ mineralization efficiency and compressive strength, simultaneous optimization of both properties was achieved to determine the optimal mixture parameters. The research findings provide a theoretical basis and design pathway for developing economically viable carbon capture and solid waste recycling technologies, which is expected to promote high-value utilization of industrial solid wastes and contribute to the achievement of carbon neutrality goals.

## 2. Materials and Methods

### 2.1. Experimental Raw Materials and Chemical Composition

**Figure 1:**
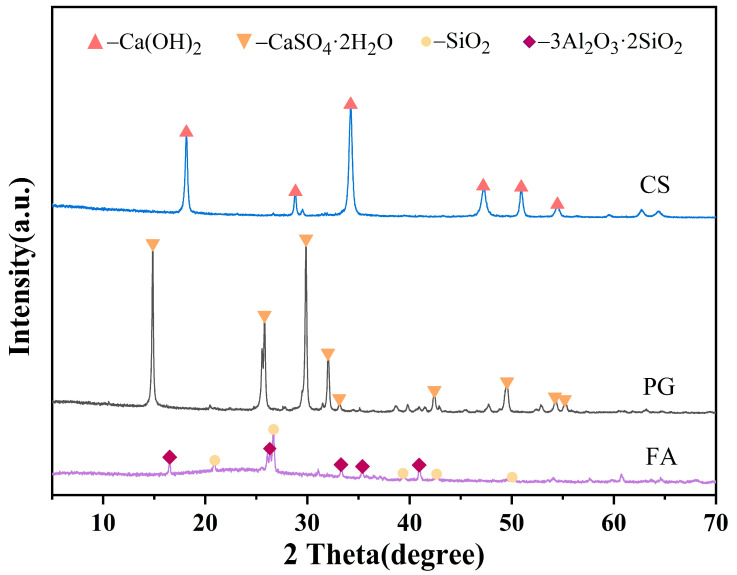
XRD patterns of the raw materials.

This study utilized three industrial solid wastes as the primary raw materials. All raw materials were dried in an oven at 105 °C for 24 h [35] before use to remove free moisture. The chemical compositions of the raw materials are listed in Table 1, while Figure 1 illustrates their mineral phase compositions. FA is mainly composed of silica–alumina components and exhibits pozzolanic properties, with minor crystalline phases of quartz and mullite. PG primarily consists of 54.89 wt% CaO and 38.64 wt% SO_3_, with XRD analysis confirming its crystalline phase as CaSO_4_·2H_2_O. CS contains a high CaO content of 95.88 wt%, predominantly present as Ca(OH)_2_, along with a small amount of CaCO_3_ (<2 wt%) formed by atmospheric carbonation. The alkaline activators used were analytically pure sodium hydroxide and industrial-grade water glass. The modulus of water glass was 3.3, and its solid content was 38%. Laboratory tap water was employed throughout the experiments.

### 2.2. Experimental Design

Response Surface Methodology (RSM) is a statistical and mathematical approach used to model processes and investigate how different input factors influence system outputs or responses [36]. This study employs the Box–Behnken Design (BBD) for RSM modeling, which maximizes information acquisition with minimal experimental points and is particularly suitable for optimization modeling of systems with three or more factors. The research systematically examines four key factors-CS content, PG content, water–solid ratio, and alkali content—that impact compressive strength and the performance of mineralization products. The level designs for these four factors are shown in Table 2.

The experiment was designed with 24 experimental points and 5 replicates at the center point to estimate the error. The selected response values were the 7-day compressive strength of the mineralization product and the CO_2_ mineralization efficiency. The sample preparation process was conducted as follows: First, the three solid raw materials were accurately weighed according to the designed mix proportions and placed in a planetary mixer. They were dry-mixed at a low speed of 60 rpm for 3 min to ensure homogeneity. Subsequently, solid sodium hydroxide was dissolved in a calculated amount of deionized water, after which water glass was added. The mixture was manually stirred for 2 min to prepare the composite alkali activator solution. While maintaining low-speed agitation in the mixer, the alkali solution was poured uniformly into the dry mixture. The mixing continued at low speed for another 3 min to ensure a homogeneous paste free of agglomerates. The fresh paste was cast into triplicate steel molds (40 mm × 40 mm × 120 mm) in two layers, compacted on a vibrating table, and leveled with a scraper. Upon completion of casting, the specimens, together with the molds, were placed in a constant temperature and humidity curing chamber. They were pre-cured for 24 h at 20 ± 2 °C and a relative humidity of 90 ± 5%. After demolding, the specimens were immediately transferred to a standard carbonation chamber for accelerated carbonation curing. The carbonation conditions were strictly controlled at 20 ± 2 °C and 60 ± 5% relative humidity. Pure CO_2_ gas (99% purity) was introduced at a constant flow rate of 0.1 L/min. Electronic valves and CO_2_ concentration sensors were utilized to maintain the CO_2_ concentration at 20%, while the pressure was maintained at atmospheric pressure.

### 2.3. Testing and Characterization Methods

#### 2.3.1. Phase Analysis

X-ray diffraction (XRD) tests were performed using a Rigaku Smartlab X-ray diffractometer (Shimadzu Instrument Co., Ltd., Kyoto, Japan) with a copper target, scanning from 5° to 70° (2θ) at a scanning rate of 5°/min. Qualitative phase identification was carried out by matching with database cards. This technique was selected due to its high specificity in identifying crystalline phases, which is critical for clarifying the formation of new phases during the mineralization process. The relative contents of specific crystalline phases were semi-quantitatively estimated using the Rietveld refinement method.

Thermogravimetric analysis (TG) was performed using an SDT Q600 thermal analyzer (TA Instruments, New Castle, DE, USA). The TG analysis was conducted on approximately 10 mg of powder, which was heated from room temperature to 1000 °C at a heating rate of 10 °C/min under a nitrogen atmosphere. The TG curves were used to quantify the content of hydration products, which exhibit dehydration peaks around 200–400 °C, and carbonates, which show decomposition peaks between 600 and 800 °C [37], which is critical for calculating CO_2_ sequestration efficiency. The mineralization efficiency calculation [38] is based on the mass loss of the sample resulting from CO_2_ release.

#### 2.3.2. Microstructure Characterization

SEM analysis was conducted using a German Zeiss Gemini 300 (Carl Zeiss AG, Germany) field emission scanning electron microscope equipped with EDS, operating at an accelerating voltage of 15 kV, to observe the microstructure, morphology, and spatial distribution of reaction products in the carbonate samples. Prior to observation, fractured surfaces of the samples were sputter-coated with gold to enhance conductivity. EDS point analysis and elemental mapping were performed to determine local chemical compositions and the distribution of elements, providing evidence for the formation of phases such as calcium carbonate and calcium silicate hydrate gel.

FT-IR analysis was performed using a Thermo Nicolet IS5 FTIR (Thermo Fisher Scientific, Waltham, MA, USA) spectrometer equipped with an attenuated total reflection (ATR) accessory to characterize the functional groups and chemical bonds present in the samples. Spectra were recorded in the range of 4000 to 400 cm^−1^. The technique was primarily employed to identify carbonates [39,40,41] (asymmetric stretching vibration band ν_3_ around 1420–1480 cm^−1^, out-of-plane bending vibration ν_2_ around 870 cm^−1^), silicate units [42] (Si–O–Si stretching vibration around 970–1100 cm^−1^), and the presence of water molecules [43] (O–H stretching vibration around 3400–3600 cm^−1^).

#### 2.3.3. Evaluation of Physical and Mechanical Properties

X-ray computed tomography (CT) was employed in this study to non-destructively and quantitatively characterize the three-dimensional microstructure of mineralization products, with a specific focus on the geometric features and spatial distribution of pore networks [44]. The measurements were conducted using an Rmct-4000 X-ray computed tomography system (Suzhou Lima Precision Measurement Technology Co., Ltd., Suzhou, China), operated at a tube voltage of 200 kV and a tube current of 220 μA. The fundamental principle of this technique involves acquiring a series of 2D projection images of the sample at various rotational angles [45,46]. These projection sequences were subsequently reconstructed into 3D volumes using specialized data analysis software, achieving a spatial resolution of 55.4 μm. Finally, image processing algorithms, including threshold segmentation and void filling, were applied to accurately distinguish and separate the solid and pore phases.

Unconfined Compressive Strength (UCS): The unconfined compressive strength of the carbonated cubic specimens was determined using a universal testing machine to evaluate their mechanical properties. The reported UCS value for each mixture represent the average of at least three valid tests. Throughout the experiment, the loading rate was consistently maintained at a steady level of 0.5 MPa/s.

### 2.4. Statistical Analysis and Modeling

This study employed the Box–Behnken Design (BBD) within Response Surface Methodology (RSM) to construct mathematical models between key influencing factors and response values, and to optimize experimental conditions based on the models. All experiments were conducted in random order to minimize systematic errors. Experimental design and data analysis were performed using Design-Expert 13 software, and the detailed specific experimental schemes and results are provided in Table 3.

## 3. Experimental Results

### 3.1. Statistical Modeling and Optimization of Process Parameters

#### 3.1.1. Model Development and Analysis of Variance

Response surface methodology (RSM) was employed to analyze the compressive strength and mineralization efficiency results, along with model reliability assessments, as presented in Table 4 and Table 5. The coefficients of variation for these responses were determined to be 12.92% and 3.92%, respectively, validating the feasibility of the experimental approach. Statistical significance of the analytical procedures, experimental validations, and influencing factors was evaluated using *p*-values, where values between 0.05 and 0.001 indicate significance, and values below 0.001 denote highly significant effects.

Factors A, B, C, and D exhibited significant influences on the response variables, with factors A and C demonstrating significance across multiple responses. Notably, factor C exerted an extremely significant effect on compressive strength, while factors A and D showed highly significant impacts on mineralization efficiency. The interaction term AD significantly affected several responses, indicating interdependent effects between these variables. Furthermore, quadratic terms significantly influenced the responses, underscoring the non-negligible nonlinear relationships between the factors and multiple response variables. These findings collectively confirm the robustness of the regression models and the complex nature of factor interactions in the studied system.

To validate model reliability, analysis of variance was conducted for the regression models of compressive strength and mineralization efficiency. The coefficient of determination (R^2^) was used to evaluate the goodness of fit. Table 5 shows R^2^ values of 0.9660 for the compressive strength model and 0.9739 for the mineralization efficiency model, indicating that the models effectively explain the majority of response variability and exhibit high predictive accuracy. The models were externally validated by comparing the fitted values with actual experimental results, which were averaged from three replicates under the optimal conditions. The results, demonstrating the model’s accuracy, are summarized in Table 6.

Nonlinear fitting analysis confirmed that a second-order polynomial model was the most suitable for characterizing the response relationships among the variables. The regression equations for compressive strength and mineralization efficiency are given by Equations (2) and (3), respectively.

(2) $$\begin{array}{c} Y_{C}=-46.85+0.518333A+0.66B+101.91667C+0.575833D+0.00125\mathrm{AB}-0.025\mathrm{AC}-0.00325\mathrm{AD}-\\ 0.175\mathrm{BC}-0.00075\mathrm{BD}-0.15\mathrm{CD}-{0.006833A}^{2}-{0.009333B}^{2}-{99.58333C}^{2}-{0.006958D}^{2} \end{array}$$

(3) $$\begin{array}{c} Y_{M}=-102.325+2.01393A+0.4876B+186.76667C+2.1461D-0.00155\mathrm{AB}-0.36\mathrm{AC}-0.010825\mathrm{AD}+\\ 0.05\mathrm{BC}-0.004925\mathrm{BD}-0.0825\mathrm{CD}-{0.021693A}^{2}-{0.006343B}^{2}-{169.4333C}^{2}-{0.028518D}^{2} \end{array}$$

Y_C_ represents the predicted value of compressive strength, Y_M_ denotes the predicted mineralization efficiency, and A, B, C, and D correspond to the four independent variables: CS content, PG content, water-to-solid ratio, and alkali content, respectively.

#### 3.1.2. Response Surface Analysis

Based on the significant regression analysis results, three-dimensional response surface plots and two-dimensional contour plots were generated to visually illustrate the impact of interactions between independent variables on the response values. The shape of the contours effectively reflects the strength of the interactions: elliptical contours indicate a significant interaction between two variables, while circular contours suggest weaker interactions.

**Figure 2:**
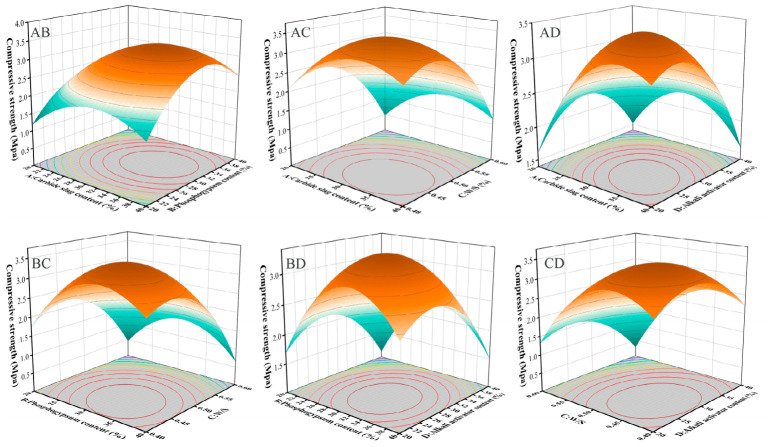
Effects of interactions between factors on compressive strength.

Figure 2 illustrates the interactive effects of various factors on compressive strength, identifying PG content and alkali dosage as the key influencing factors. The compressive strength declines when either the PG content or alkali content deviated from the optimal range. Excessive PG may introduce surplus sulfate ions, thereby interfering with the formation of silicate gel phases, whereas insufficient alkali can lead to incomplete activation of the precursors. To achieve optimal mechanical performance, it is recommended to maintain the PG content between 29% and 32% and the alkali content between 27% and 30%. This range likely represents a balanced state where the alkali content sufficiently activates the aluminosilicate source, while the PG provides beneficial sulfate ions to promote strength development.

**Figure 3:**
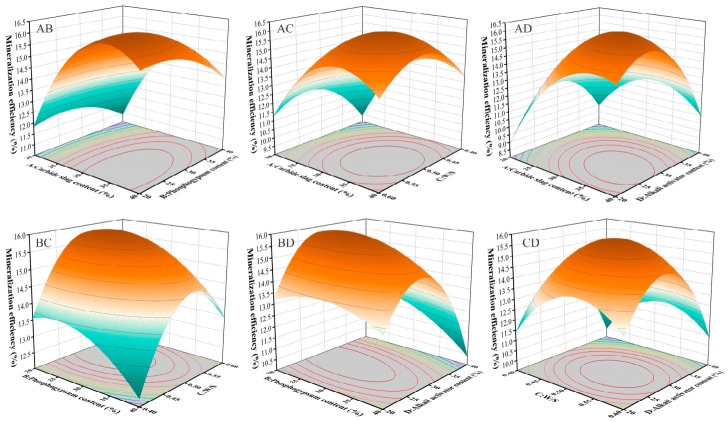
Effects of interactions between factors on mineralization efficiency.

Figure 3 presents the interactive effects of various factors on carbonation efficiency, with CS content and alkali content playing dominant roles. As the primary calcium source for carbonation, CS directly determines the theoretical CO_2_ absorption capacity. The critical role of alkali content is attributed to its regulation of the system’s pH, which in turn governs the dissolution rate of calcium species and the precipitation kinetics of carbonate phases. To optimize the carbon sequestration capacity of the material, it is recommended to control the CS content between 33% and 37%, and the alkali content between 29% and 32%. This parameter combination helps balance reactivity and stability, thereby enhancing the overall environmental benefits. Specifically, this alkali range ensures a sufficiently high pH to drive a rapid carbonation process, without being so high as to cause excessive silicate polymerization.

#### 3.1.3. Model Verification and Multi-Response Optimization

Analysis of variance and multi-objective optimization of the response surface models were performed using Design-Expert software, with the compressive strength and mineralization efficiency of the material as the response variables, to determine the optimal formulation parameters. The model predictions indicated that the optimal formulation consisted of 34% CS content, 30% PG content, a water-to-solid ratio of 0.48, and 27% alkali content. As shown in Table 6, under this formulation, the predicted values of compressive strength and mineralization efficiency closely matched the experimentally measured values, with relative errors all below 5%, demonstrating significant predictive reliability of the models. These results validate the effectiveness of the response surface methodology in optimizing formulations for multi-component systems and provide a theoretical basis for the design of similar cementitious materials.

### 3.2. Effects of Various Factors on Compressive Strength and Mineralization Efficiency

**Figure 4:**
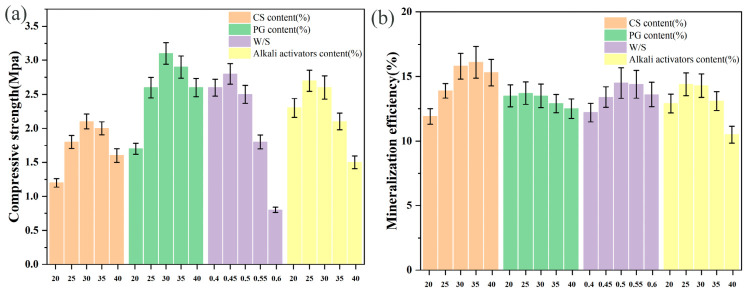
Effects of single factors on materials: (**a**) compressive strength, (**b**) mineralization efficiency. Single-factor analysis conducted by varying one parameter while keeping others at their central values (CS: 30 wt%, PG: 25 wt%, W/S: 0.45, Alkali activators content: 25 wt%).

To intuitively demonstrate the influence trend of a single key factor, we conducted a series of gradient experiments. This approach was based on the optimal parameter combination determined by Response Surface Methodology (RSM), as shown in Table 6. In these experiments, the target factor was varied across a series of gradients, while all other factors were held constant at their respective optimal levels. The relevant results are presented in Figure 4. When the dosages of CS and PG exceeded 25%, the material compressive strength showed a significant increase. This is primarily attributed to the highly reactive Ca(OH)_2_ provided by CS, which not only directly participates in carbonation reactions to form CaCO_3_ crystals but also creates a strongly alkaline environment (pH > 12) that effectively stimulates the dissolution of reactive SiO_2_ and Al_2_O_3_ in fly ash, promoting the formation of cementitious products such as C-S-H and AFt. Excess water leads to excessive internal porosity, disrupting the network continuity of C-S-H—an effect that becomes particularly pronounced when the water-to-solid ratio exceeds 0.5, resulting in a sharp decrease in compressive strength.

For mineralization efficiency, the influence intensity of PG dosage and water-to-solid ratio was significantly weaker than that of CS content and alkali content. This difference mainly stems from the inherent characteristic of PG requiring energy-intensive pretreatment to release effective calcium sources, while the water-to-solid ratio primarily affects mass transfer rates rather than the reaction driving force. The growth of mineralization efficiency slowed after CS dosage exceeded 30%, a phenomenon closely related to product layer mass transfer resistance: as CS content increases, the concentration of active Ca^2+^ in the system rises significantly, accelerating CO_2_ mineralization reaction kinetics. However, excessively rapid surface reactions form dense, low-permeability carbonate coating layers that hinder further participation of core materials in mineralization reactions.

### 3.3. Evolution of Phase Composition

**Figure 5:**
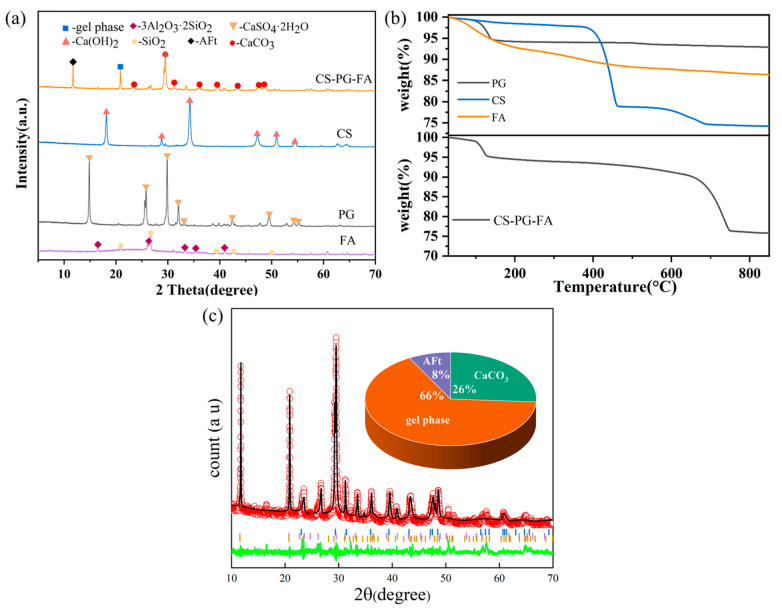
Phase evolution of CS-PG-FA system (**a**) XRD comparison of raw materials and CS-PG-FA system, (**b**) TG comparison of raw materials and CS-PG-FA system, (**c**) Rietveld-refined XRD results of CS-PG-FA.

Figure 5a presents the comparative XRD patterns of raw materials and the CS-PG-FA system after mineralization. CaSO_4_·2H_2_O in PG was transformed into ettringite (AFt, 2θ = 10.5°) with only minor residual CaSO_4_ remaining. The characteristic peaks of Ca(OH)_2_ (2θ = 18.0°, 34.0°) from carbide slag were nearly absent, corresponding to the formation of CaCO_3_ (2θ = 29.4°). The reduced intensity of the broad diffraction peak (2θ = 20–35°) for the silicoaluminate glass phase in fly ash indicates its participation in geopolymerization to form calcium silicate hydrate gel. As shown in Figure 5c, the XRD fitting results further indicate that the crystalline phases of the FA-CS-PG products primarily consisted of 66% gel phase, 26% CaCO_3_, and 8% AFt.

Figure 5b TG analysis further validates these transformations: raw PG exhibits significant mass loss (≈15.7%) between 100 and 200 °C, corresponding to the stepwise dehydration of CaSO_4_·2H_2_O, confirming its calcium source requires liberation before participating in subsequent reactions. Carbide slag shows a Ca(OH)_2_ dehydration peak at 400–500 °C and an overlapping CaCO_3_ decomposition peak at 600–800 °C, indicating the coexistence of free Ca(OH)_2_ and carbonate impurities in its original composition. After mineralization, the CS-PG-FA system displayed the disappearance of the 400–500 °C Ca(OH)_2_ characteristic peak, confirming complete conversion of reactive calcium from carbide slag into carbonates.

### 3.4. Molecular Structure Evolution: FT-IR

**Figure 6:**
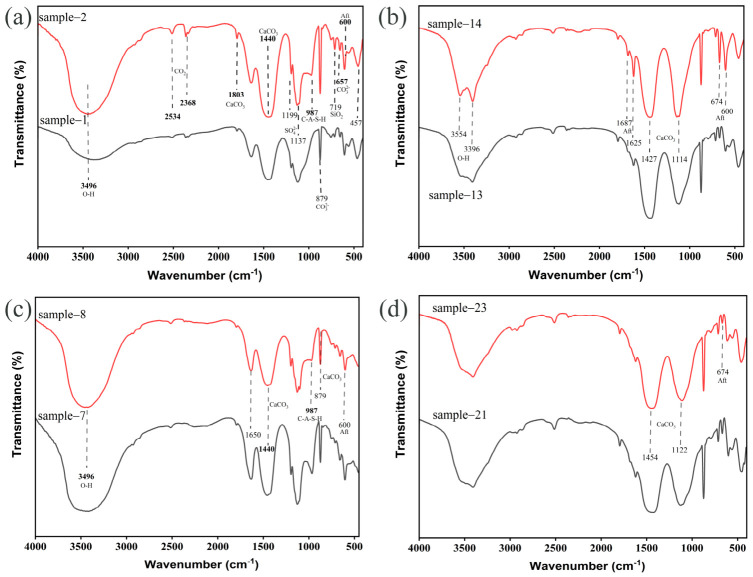
Effects of various factors on phase transformation (**a**) CS content: 1–20%, 2–40%, (**b**) PG content: 13–20%, 14–40%, (**c**) w/s: 7–0.4, 8–0.6, (**d**) Alkali content: 21–20%, 23–40%.

As the primary calcium source, CS predominantly supplies Ca(OH)_2_ to elevate system alkalinity and increase available Ca^2+^ for mineralization. Figure 6a demonstrates that increasing CS content enhances the intensity of calcium carbonate characteristic peaks at 1803 cm^−1^ (ν_2_ out-of-plane bending) and 1446 cm^−1^ (ν_3_ asymmetric stretching of CO_3_^2−^), indicating promoted CaCO_3_ crystallization. Concurrently, intensified CO_2_ adsorption peaks at 2534 cm^−1^ (ν_2_ bending) and 2368 cm^−1^ (ν_3_ asymmetric stretching) suggest incomplete CO_2_ conversion, likely caused by excessive Ca(OH)_2_ accelerating mineralization—this rapid reaction generates surface-covering products that impede further reaction progression. Additionally, enhanced peak intensities at 987 cm^−1^ (Si–O stretching of C–A–S–H) and 600/657 cm^−1^ (AFt) confirm CS-derived Ca^2+^ concurrently activates pozzolanic reactions and promote cementitious phase formation.

PG supplies SO_4_^2−^, which is a key component for the crystallization of AFt. As shown in Figure 6b, with increasing PG content, the intensity of the characteristic peaks of AFt at 600 cm^−1^, 674 cm^−1^, and 1625 cm^−1^ is enhanced. It is worth noting that the PG content had no significant influence on the peak intensity of calcium carbonate at 1446 cm^−1^.

The water-to-solid ratio primarily regulates ion diffusion and the nucleation kinetics of the cementitious phases. A decrease in the water-to-solid ratio increases ion concentration in the system, accelerating the heterogeneous nucleation and growth of C-A-S-H and AFt. As shown in Figure 6c, with the reduction in the water-to-solid ratio, the band intensities of AFt (674 cm^−1^) and C-A-S-H (971 cm^−1^, 1650 cm^−1^) are enhanced.

Alkali content significantly influences phase evolution through OH^−^ concentration. Figure 6d illustrates that as alkali content increases, the peak at 674 cm^−1^ (characteristic bending vibration of SO_4_^2−^) weakens, indicating suppressed AFt formation. Concurrently, the peak at 1122 cm^−1^ (Si–O–Si) sharpens, reflecting an increased polymerization degree of C–A–S–H. This phenomenon occurs because high OH^−^ concentration promotes the formation of stable Al(OH)_4_^−^ complexes from Al^3+^, which inhibits the reactions required for AFt generation. Simultaneously, it accelerates the dissolution of SiO_2_, enhancing the polymerization degree of C–A–S–H.

### 3.5. X-CT

**Figure 7:**
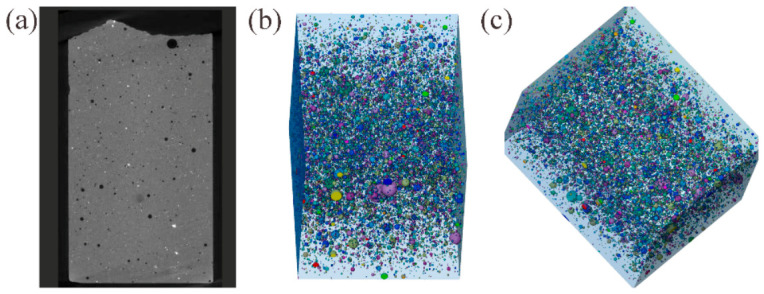
3D Pore Structure of CS-PG-FA System After Mineralization: (**a**) Original X-CT scan image; (**b**) Front view of 3D-CT reconstructed image; (**c**) Tilted view of 3D-CT reconstructed image.

X-ray computed tomography (X-CT) was employed to visualize and quantify the evolution of pore structure during the CO_2_ mineralization process. The raw CT projections were reconstructed using Avizo 2019 software, followed by threshold segmentation based on grayscale histogram analysis to distinguish pores from solid phases. As shown in Figure 7b,c, the three-dimensional reconstructed pore network reveals a gradient distribution of pore sizes along the material depth. The surface region is dominated by smaller pores, while the inner region contains some larger pores. This gradient confirms an outside-in mineralization progression: CO_2_ diffuses from the surface and reacts with Ca^2+^ to form CaCO_3_ crystals, which preferentially fill smaller pores, leaving larger connected pores in the unreacted interior. The pore maps derived from CT provide critical insights for optimizing mineralization efficiency. The presence of large internal pores indicates incomplete CO_2_ diffusion, which could be improved by enhancing ion mobility to refine the material’s microstructure. This offers guidance for designing materials that balance carbon sequestration capacity with mechanical strength.

### 3.6. Microstructure and Morphology: SEM-EDS Analysis

**Figure 8:**
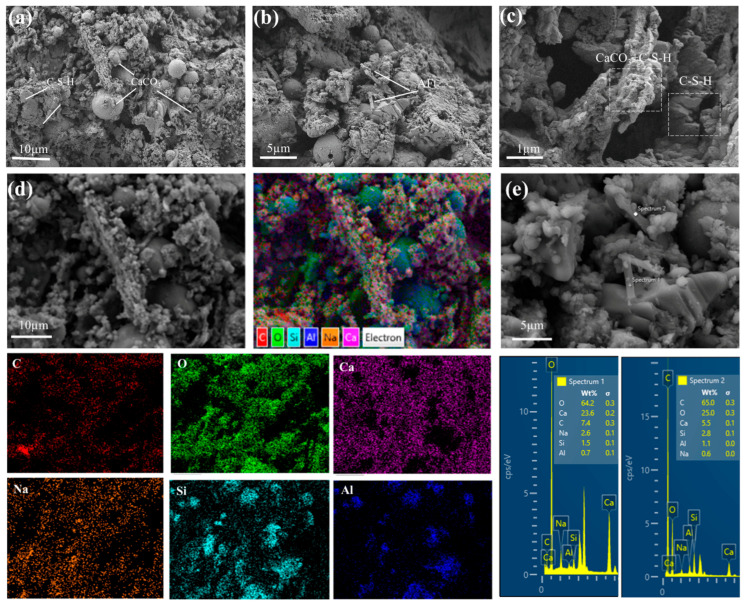
SEM Image of Samples After 7 Days of Mineralization (**a**) ×1K, (**b**) ×2K, (**c**) ×10K, (**d**) Surface element distribution, (**e**) Energy spectrum on surface.

To elucidate the relationship between microstructure and mechanical properties, SEM analysis was performed on the specimens with optimal performance, as shown in Figure 8. The micrographs reveal that the carbonated material formed after mineralization consists of two primary hydration products: low-crystallinity calcium silicate hydrate gel and ettringite. Notably, distinct pores are observable on the surface, which may account for the relatively lower early-age compressive strength. However, these pores also serve as essential channels for gas transport during the mineralization process. X-CT results indicate a gradient distribution of pore sizes along the material depth: the surface region is dominated by smaller pores, while larger, interconnected pores exist in the interior. This gradient structure confirms an “outside-in” mineralization process. Consequently, these surface and near-surface pores provide the necessary pathways for initial CO_2_ diffusion, facilitating the penetration of reactive gas into the material’s interior. Nevertheless, the persistence of larger internal pores identified by X-CT indicates inherent transport limitations. These internal voids represent zones of incomplete CO_2_ diffusion and reaction, ultimately restricting the overall mineralization efficiency. As shown in Figure 8b, the FA particles exhibit limited reactivity, retaining their characteristic spherical morphology with only localized etching on the glassy surface, thus acting primarily as inert fillers and nucleation sites for hydration products. FA particles exhibit limited reaction extent, retaining their characteristic spherical morphology with only localized erosion on the glassy surfaces as shown in Figure 8b, primarily acting as inert fillers and nucleation sites for hydration products.

To investigate the role of each component in the mineralization process, SEM elemental mapping and EDS spectral analysis were conducted. The elemental distribution maps further clarified the spatial distribution of reactants and products. Signals of Ca and Na were uniformly dispersed, corresponding to the homogeneous mixing of CS and alkali activators. In contrast, carbon enrichment zones were primarily concentrated in CS-rich regions, indicating that mineralization products preferentially formed in areas with the highest concentration of Ca^2+^. This confirms that CS, with its high CaO content, serves as the main source of Ca^2+^ for the carbonation reaction. Quantitative EDS analysis of the mineralized regions revealed a Ca:C atomic ratio of 3.2, which aligns closely with the theoretical ratio of 3.3 [47] for pure calcite. Combined with the dominant calcite characteristic peak (2θ = 29.4°) observed in the XRD results, it is confirmed that CaCO_3_ is the primary mineralization product.

In summary, the synergistic mechanism of this system is as follows: CS serves as the core calcium source, where free Ca(OH)_2_ directly mineralizes to form CaCO_3_ while providing a strongly alkaline environment (pH > 12) to promote the dissolution of aluminosilicate glass phases in FA. FA contributes active SiO_2_ and Al_2_O_3_ that react with Ca(OH)_2_ to generate amorphous gels, which construct a three-dimensional skeleton and alleviate mass transfer resistance caused by CaCO_3_ coating layers. PG functions as a regulator by supplying SO_4_^2−^ ions to promote ettringite formation for early strength enhancement, while its dehydrated product CaSO_4_ modulates CaCO_3_ crystal morphology through ion competition.

## 4. Discussion

This study, through RSM optimization, identified specific compositional ratios within the FA-CS-PG ternary solid waste system that enable the synergistic optimization of CO_2_ mineralization efficiency and 7-day compressive strength. These findings present a potential technical pathway for the high-value resource recovery of industrial solid wastes coupled with carbon capture. However, transitioning from idealized laboratory research to complex real-world engineering applications necessitates more rigorous, systematic, and critical assessments of environmental risks, practical applicability, and inherent technological limitations.

First, a thorough evaluation of potential environmental risks across the entire lifecycle is essential. Microstructural analyses indicate the formation of a dense reaction matrix. Nevertheless, as industrial by-products, FA, CS, and PG typically contain varying concentrations of heavy metals and other potential contaminants. The current study has not sufficiently validated whether the mineralized products effectively immobilize these pollutants or their long-term stability and leaching behavior under realistic environmental conditions. This represents a significant limitation of the present work. Subsequent research should prioritize standard leaching toxicity tests and accelerated aging experiments to provide empirical evidence supporting the environmental safety of this technology.

Second, the 7-day compressive strength of approximately 3.5 MPa achieved in this study suggests that the most direct and appropriate engineering applications should target non-load-bearing or low-load-bearing structural contexts. In the industrial-scale trial of “synergistic mining-filling paste backfill” conducted at Renjiazhuang Coal Mine in Ningdong Mining Area, Ningxia, a low-water-content coal-based solid waste backfill material was developed through Response Surface Methodology (RSM) optimization. The material achieved a 28-day uniaxial compressive strength ranging from 1.7 to 3.5 MPa and was successfully applied to goaf filling in the mining face, effectively enabling the synergistic utilization of bulk solid waste. The backfill plant demonstrated an annual operational capacity of 300,000 tons per year.

Finally, the conclusions of this study are based on laboratory conditions featuring homogeneous raw materials, precisely controlled mix proportions, and constant curing environments. Industrial-scale deployment will face challenges such as batch-to-batch variability in feedstock composition, heterogeneity during large-scale mixing and construction, and unpredictable ambient conditions—all of which may compromise product performance stability and the anticipated synergistic effects. Moreover, the reported 7-day mineralization efficiency primarily reflects the rapid early-stage reaction phase; the long-term cumulative carbon sequestration capacity remains uncertain. As the reaction progresses, the diffusion rate of CO_2_ through the increasingly dense carbonate layer is expected to decline. These uncertainties related to raw materials, processing, and long-term performance represent critical constraints in evaluating the scalability and techno-economic feasibility of the technology.

## 5. Conclusions

Using RSM, this study systematically elucidated how raw material proportions and process parameters regulate the FA-CS-PG ternary system. Under controlled laboratory conditions, the optimized parameters were identified as 34% CS, 30% PG, W/S of 0.48, and 27% alkali content. This formulation yielded a compressive strength of 3.5 MPa and a 16% CO_2_ mineralization efficiency after 7 days. Notably, this “optimal” result—derived from homogeneous materials and ideal curing—serves primarily to verify the system’s theoretical potential for synergistic strengthening, rather than defining a ready-made industrial formulation.

Mechanistically, the components exhibit distinct functional roles: CS acts as a calcium source and alkali activator, driving calcium carbonate formation and disrupting the FA glassy phase; FA contributes to an aluminosilicate network via pozzolanic reactions; and PG promotes ettringite formation to fill pores and enhance early strength. This synergy balances strength development with CO_2_ mineralization.

However, scaling this research to industrial implementation presents significant challenges. First, the 3.5 MPa compressive strength limits current applications to non-load-bearing or low-load-bearing structures. Second, scalability remains a hurdle; large-scale production may face issues with mixing uniformity, heat transfer, and CO_2_ diffusion. Furthermore, raw material variability poses a major limitation, as the significant batch-to-batch fluctuations in industrial-grade FA, CS, and PG differ from the specific materials used here. Finally, long-term uncertainties regarding CO_2_ sequestration stability, durability in complex environments, and potential leaching risks require assessment through long-term monitoring or accelerated aging tests.

In conclusion, this study provides a quantitative basis and theoretical support for the design of solid-waste-based mineralized materials by clarifying their synergistic mechanisms at the laboratory scale. Yet, successful industrial translation will depend on further evaluating scalability, raw material adaptability, environmental safety, and economic feasibility. Distinguishing the boundary between laboratory validation and engineering application is a crucial step toward practical implementation.

## Figures and Tables

**Table 1:** Chemical composition of the raw materials. (wt%).

Materials	CaO	SiO_2_	Al_2_O_3_	Fe_2_O_3_	MgO	SO_3_	TiO_2_	MnO_2_
FA	2.03	59.61	28.85	3.82	1.02	0.16	1.77	0.06
CS	95.88	0.51	0.938	0.06	0.34	0.30	0.03	0.07
PG	54.89	1.42	0.32	1.39	0.71	38.64	0.30	0.09

**Table 2:** Coded Factor Levels.

Independent Variable Factor	Level: −1	Level: 0	Level: 1
A, Carbide slag content	20%	30%	40%
B, Phosphogypsum content	20%	30%	40%
C, w/s	0.4	0.5	0.6
D, Alkali activators content	20%	30%	40%

**Table 3:** Response Surface Methodology.

	A: Carbide Slag Content (%)	B: Phosphogypsum Content (%)	C: W/S	D: Alkali Activator Content (%)	Compressive Strength (Mpa)	Mineralization Efficiency (%)
sample 1	20	20	0.5	30	1.3	11.23
sample 2	40	20	0.5	30	1.9	14.83
sample 3	20	40	0.5	30	1.5	10.53
sample 4	40	40	0.5	30	2.6	13.51
sample 5	30	30	0.4	20	2.8	10.86
sample 6	30	30	0.6	20	1.2	12.64
sample 7	30	30	0.4	40	2.6	9.09
sample 8	30	30	0.6	40	0.4	10.54
sample 9	20	30	0.5	20	1.7	9.05
sample 10	40	30	0.5	20	2.7	14.45
sample 11	20	30	0.5	40	1.6	9.64
sample 12	40	30	0.5	40	1.3	10.71
sample 13	30	20	0.4	30	1.4	13.84
sample 14	30	40	0.4	30	2.5	12.63
sample 15	30	20	0.6	30	0.4	14.58
sample 16	30	40	0.6	30	0.8	13.57
sample 17	20	30	0.4	30	1.9	9.77
sample 18	40	30	0.4	30	2.9	13.63
sample 19	20	30	0.6	30	0.3	11.37
sample 20	40	30	0.6	30	1.2	13.79
sample 21	30	20	0.5	20	1.8	13.44
sample 22	30	40	0.5	20	2.4	12.81
sample 23	30	20	0.5	40	1	13.21
sample 24	30	40	0.5	40	1.3	10.61
sample 25	30	30	0.5	30	3.2	15.67
sample 26	30	30	0.5	30	3.3	15.29
sample 27	30	30	0.5	30	3.1	15.91
sample 28	30	30	0.5	30	3.4	15.43
sample 29	30	30	0.5	30	3.2	15.74

**Table 4:** Regression models and analysis of variance of the corresponding model terms.

Response	Compressive Strength		Mineralization Efficiency	
	F-Value	*p*-Value	F-Value	*p*-Value
Model	28.40	<0.0001	37.31	<0.0001
A	24.77	0.0002	125.13	<0.0001
B	14.59	0.0019	18.69	0.0007
C	128.67	<0.0001	14.90	0.0017
D	25.94	0.0002	29.91	<0.0001
AB	1.00	0.3332	0.3862	0.5443
AC	0.0402	0.8440	2.08	0.1709
AD	6.79	0.0207	18.84	0.0007
BC	1.97	0.1823	0.0402	0.8440
BD	0.3617	0.5572	3.90	0.0684
CD	1.45	0.2490	0.1094	0.7457
A^2^	48.69	<0.0001	122.67	<0.0001
B^2^	90.84	<0.0001	10.49	0.0059
C^2^	103.41	<0.0001	74.83	<0.0001
D^2^	50.49	<0.0001	212.00	<0.0001
Lack of Fit		0.1446		0.0691

**Table 5:** Model reliability test analysis.

Group	R^2^	Adj R^2^	Pred R^2^	C.V./%	Adeq Precision
Model Y_C_	0.9660	0.9320	0.8250	12.92	16.8646
Model Y_M_	0.9739	0.9478	0.8579	3.92	19.3082

**Table 6:** Optimal Proportions and Results.

	A	B	C	D
Optimal ratio	34%	30%	0.48	27%
Performance	Compressive strength (Mpa)		Mineralization efficiency (%)	
Model	3.62		16.11	
Experiment	3.47		15.81	

## Data Availability

The original contributions presented in this study are included in the article. Further inquiries can be directed to the corresponding author.
